# Association of Shank 1A Scaffolding Protein with Cone Photoreceptor Terminals in the Mammalian Retina

**DOI:** 10.1371/journal.pone.0043463

**Published:** 2012-09-12

**Authors:** Salvatore L. Stella, Alejandro Vila, Albert Y. Hung, Michael E. Rome, Uyenchi Huynh, Morgan Sheng, Hans-Juergen Kreienkamp, Nicholas C. Brecha

**Affiliations:** 1 Department of Ophthalmology, University of Missouri-Kansas City, School of Medicine, Kansas City, Missouri, United States of America; 2 Department of Basic Medical Sciences, University of Missouri-Kansas City, School of Medicine, Kansas City, Missouri, United States of America; 3 Department of Medicine David Geffen School of Medicine, University of California Los Angeles, Los Angeles, California, United States of America; 4 Department of Neurobiology, David Geffen School of Medicine, University of California Los Angeles, Los Angeles, California, United States of America; 5 Jules Stein Eye Institute, David Geffen School of Medicine, University of California Los Angeles, Los Angeles, California, United States of America; 6 CURE Digestive Diseases Research Center, VAGLAHS, Los Angeles, California, United States of America; 7 Veterans Affairs Greater Los Angeles Healthcare System, Los Angeles, California, United States of America; 8 Institut für Humangenetik, Universitätsklinikum Hamburg-Eppendorf, Martinistrasse 52, Hamburg, Germany; 9 The Picower Institute for Learning and Memory, The Institute of Physical and Chemical Research (RIKEN)-Massachusetts Institute of Technology Neuroscience Research Center, Howard Hughes Medical Institute, Massachusetts Institute of Technology, Cambridge, Massachusetts, United States of America; 10 Department of Neurology, Massachusetts General Hospital, Boston, Massachusetts, United States of America; University of Oldenburg, Germany

## Abstract

Photoreceptor terminals contain post-synaptic density (PSD) proteins e.g., PSD-95/PSD-93, but their role at photoreceptor synapses is not known. PSDs are generally restricted to post-synaptic boutons in central neurons and form scaffolding with multiple proteins that have structural and functional roles in neuronal signaling. The Shank family of proteins (Shank 1–3) functions as putative anchoring proteins for PSDs and is involved in the organization of cytoskeletal/signaling complexes in neurons. Specifically, Shank 1 is restricted to neurons and interacts with both receptors and signaling molecules at central neurons to regulate plasticity. However, it is not known whether Shank 1 is expressed at photoreceptor terminals. In this study we have investigated Shank 1A localization in the outer retina at photoreceptor terminals. We find that Shank 1A is expressed presynaptically in cone pedicles, but not rod spherules, and it is absent from mice in which the Shank 1 gene is deleted. Shank 1A co-localizes with PSD-95, peanut agglutinin, a marker of cone terminals, and glycogen phosphorylase, a cone specific marker. These findings provide convincing evidence for Shank 1A expression in both the inner and outer plexiform layers, and indicate a potential role for PSD-95/Shank 1 complexes at cone synapses in the outer retina.

## Introduction

Postsynaptic density (PSD) protein-95 family members (e.g., PSD-95, PSD-93) are associated with presynaptic photoreceptor terminals in the outer plexiform layer (OPL) in the retina [1]. However, the functional role of PSD-95 family members in photoreceptor terminals is not known. In central neurons, PSD-95 family members are associated with post-synaptic sites and linked to multiple anchoring/scaffold proteins [2], [3]. PSD-95 family members interact with a variety of signaling and cytoskeletal proteins, including, the Shank family of proteins, which are reported to function as putative anchoring proteins for PSD-95, ionotropic and metabotropic glutamate receptors, and L-type Ca^2+^ channels in neurons [4], [5], [6], [7], [8].

Shank proteins consist of 3 major family members: (Shank 1-3) (Sheng and Kim 2000), and contain five domain/regions that are involved in protein-protein interactions: 1) ankyrin repeats, 2) SH3 domain, 3) PDZ domain, 4) a proline-rich region and 5) SAM domain [8]. The PDZ domain of Shank directly interacts with the C-terminal QTRL motif of GKAP/SAPAP/DAP-1 [8], [9], a protein that binds to the GK domain of the PSD-95 family of proteins [10], [11], [12], [13], [14], [15], [16]. The Shank family of proteins has been shown to interact with group I metabotropic glutamate receptors via the Homer1 protein, which in turn interacts with IP_3_Rs [9], [17], [18], [19], [20]. Shank proteins also interact with several actin regulatory proteins [21], and L-type Ca^2+^ (Ca_V_1.3) channels in medium spiny neurons of the striatum [4], [22], [23]. Shank 1 expression is restricted to neurons [24], including retina [25], except during embryonic development in *Xenopus* where Shank 1 transcripts have been observed with *in situ* hybridization in the pronephros [25]. Alternative splicing of Shank 1 has been shown to yield multiple Shank 1 isoforms. The Shank 1A isoform has all five functional domain regions (ankyrin, SH3, PDZ, proline-rich, and SAM), and it is localized to excitatory synapses with PSD-95 [26]. Since photoreceptors possess a unique and yet undefined role for PSDs, it is possible that Shank 1A is expressed at photoreceptor synapses and may contribute to photoreceptor signaling at the terminal.

To address this question we used a transgenic line expressing yellow fluorescent protein (YFP) under control of the *thy*-1.2 promoter that labels cone bipolar cells in the retina [27]. The *thy*-1.2 YFP-expressing mouse cone bipolar cells allows us to easily differentiate presynaptic expression of proteins in the outer retina at photoreceptor terminals from postsynaptic expression of proteins expressed at cone bipolar cell dendrites in the mouse retina at the light microscopic level. Our findings indicate that Shank 1A is expressed at both synaptic layers of the retina, in the OPL shank1 A is restricted solely to cone pedicles and is absent from rod spherules, in the IPL, Shank 1A is homogenously expressed throughout the synaptic layer, likely present at amacrine and ganglion cell processes where it can assemble with the postsynaptic complex. Thus, this differential expression of Shank 1A in the outer retina at photoreceptor terminals may influence cone signaling, and account for some of the reported differences in the output properties of rod and cone photoreceptors.

## Results

### Shank 1A is expressed in both the OPL and the IPL

**Figure 1:**
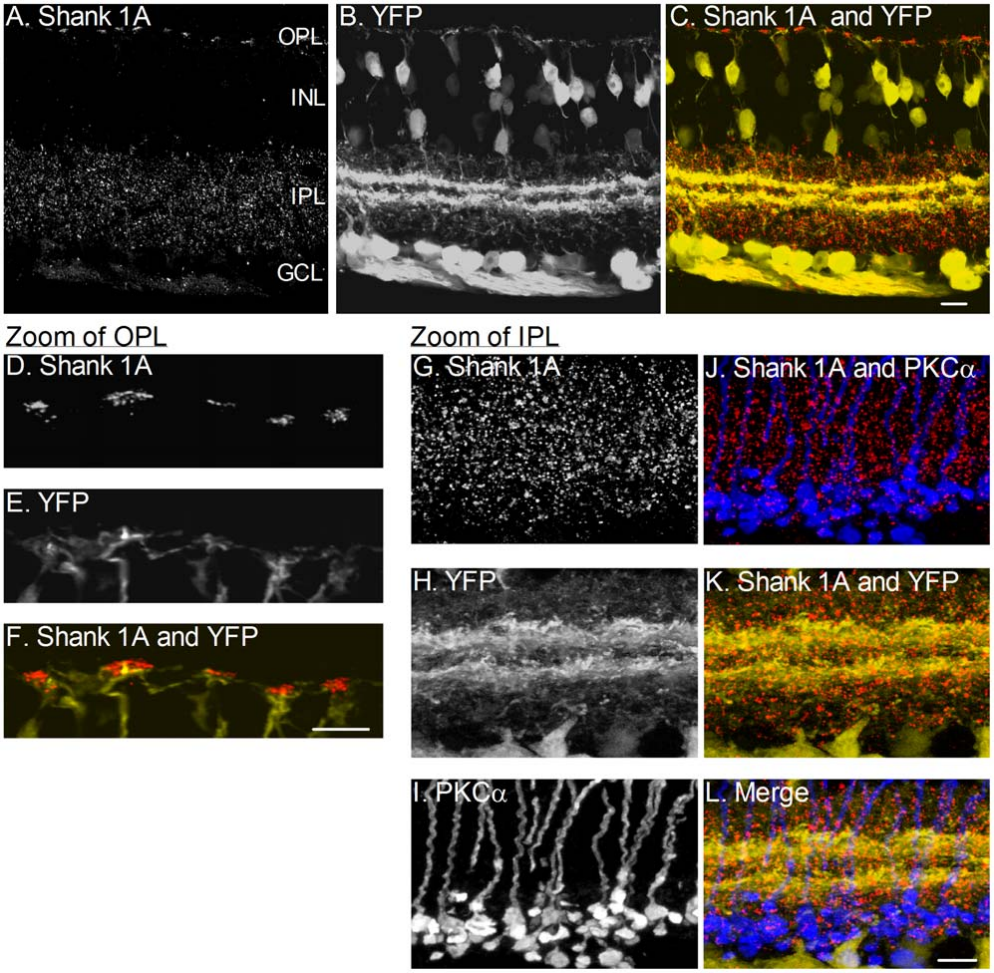
Shank 1A immunoreactivity is in both the inner plexiform layer (IPL) and outer plexiform layer (OPL) of the mouse YFP-16 line retina. **A–C**: A. Image of a retinal section immunostained for Shank 1A. B. Mouse YFP-16 line vertical retinal section. C. Shank 1A (red) immunolabeling and YFP (yellow). Shank1A expression is restricted to the OPL and IPL. A regular pattern of Shank 1A immunolabeling appears in the OPL, which is indicative of cone photoreceptor terminals. **D–E**: High magnification zoom of the OPL demonstrates that Shank 1A puncta (red) are distal to the dendrite tips (yellow) of YFP labeled cone bipolar cells, suggesting that Shank 1A is expressed presynaptic to the YFP cone bipolar cell dendrite. **G–L**: High magnification zoom of the IPL demonstrates that Shank 1A puncta are likely expressed postsynaptically to bipolar cell terminals. G. Shank 1A immunoreactive puncta. H. YFP labeled neurons and processes within the IPL region. I. PKCα labeled rod bipolar cell axons and terminals. J. Combined Shank 1A (red) and PKCα (blue) immunolabeling illustrate that shank 1A puncta are postsynaptic to rod bipolar cell terminals in the IPL. K. Combined Shank 1A (red) immunolabeling and YFP (yellow) in the IPL demonstrate that Shank 1A puncta are postsynaptic to cone bipolar cell terminals in the IPL. L. Combined triple fluorescent image of Shank 1A (red), PKCα (blue), and YFP (yellow) in the IPL. OPL = outer plexiform layer, INL = inner nuclear layer, IPL = inner plexiform layer, and GCL = ganglion cell layer. Scale bars = 10 µm.

Immunostaining of vertical sections of the adult mouse (*thy*-1.2 YFP 16 line) retina with antisera against Shank 1A showed Shank 1A immunoreactivity in the outer plexiform layer (OPL) and the inner plexiform layer (IPL) of the retina (Fig. 1A–C). All three Shank 1 antibodies used in this study produced similar punctate immunolabeling in the OPL and the IPL of the mouse retina. These antibodies have been previously characterized in neurons using Western blotting and immunohistochemistry [6], [24], [28]. The YFP-16 mouse line contains the YFP reporter signal in cone bipolar cells, amacrine cells, and ganglion cells (see Fig. 1B, [27]). Shank 1A immunofluorescence had a punctate appearance in both plexiform layers, suggesting a synaptic localization. In the OPL, Shank 1A immunoreactivity consisted of large clusters at the base of the OPL that are indicative of cone pedicles (Fig. 1D–F). Each cluster of Shank 1A puncta were in close apposition and distal to the tips of the dendrites of YFP cone bipolar cells (Fig. 1F), suggesting that Shank 1A was restricted to the presynaptic cone terminal, and not present in cone bipolar cell dendrites. Shank 1A-immunoreactive puncta were homogeneously distributed throughout the IPL (Fig. 1G–L), and found around YFP processes (Fig. 1K) and PKCα immunoreactive axons and terminals (Fig. 1J) in the IPL.

**Figure 2:**
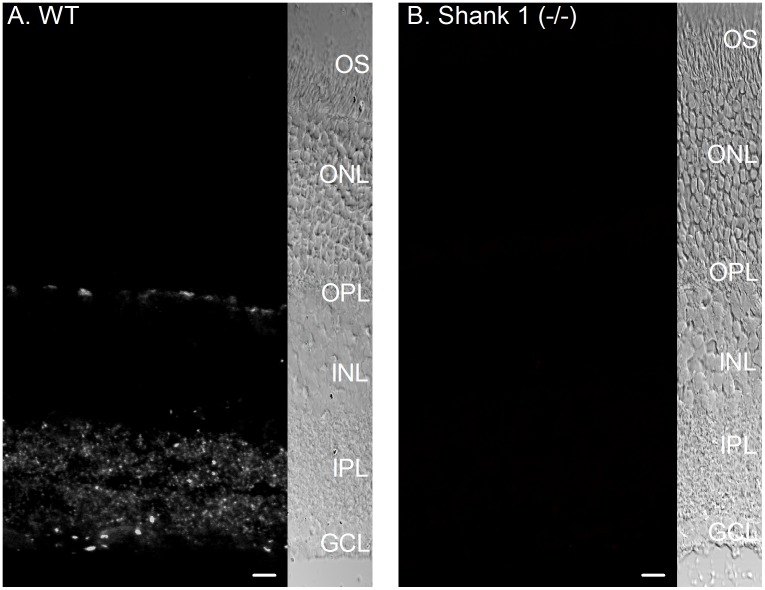
Shank 1A immunoreactivity is absent when the Shank 1 gene has been deleted in mouse retina. **A**: Shank 1A immunoreactivity in wild type mouse retina, punctate antibody labeling in the OPL in the outer retina and throughout the IPL. **B**: No Shank 1A immunoreactivity was found in retinal sections obtained from animals where the Shank 1 gene was deleted. (OS = outer segment, ONL = outer nuclear layer, OPL = outer plexiform layer, INL = inner nuclear layer, IPL = inner plexiform layer, and GCL = ganglion cell layer). Scale bar is 10 µm.

Several lines of evidence suggest that the immunostaining is specific for Shank 1A. First, in all previous studies characterizing Shank 1 antibodies, immunoblotting of wild-type mouse brain extracts with Shank 1 antibodies showed a major band in the 240–288 kDa range [24], [29], [30]. In addition, this band was decreased in the heterozygote and eliminated in the homozygous shank 1-/- knockout mouse brain [29]. Second, the same immunolabeling pattern was obtained using three different antibodies (see Methods). Third, the immunostaining for all three antibodies used in this study were absent in retinal sections obtained from mice were the Shank 1 gene was deleted (one example is shown; see Fig. 2). However, expression of other prominent synaptic proteins, like CTBP2/Ribeye and PSD-95 were not disrupted in the outer retina and within the OPL of mice where the Shank 1 gene had been deleted (Fig. S1).

### Shank 1A and PSD-95 are clustered together in the OPL

**Figure 3:**
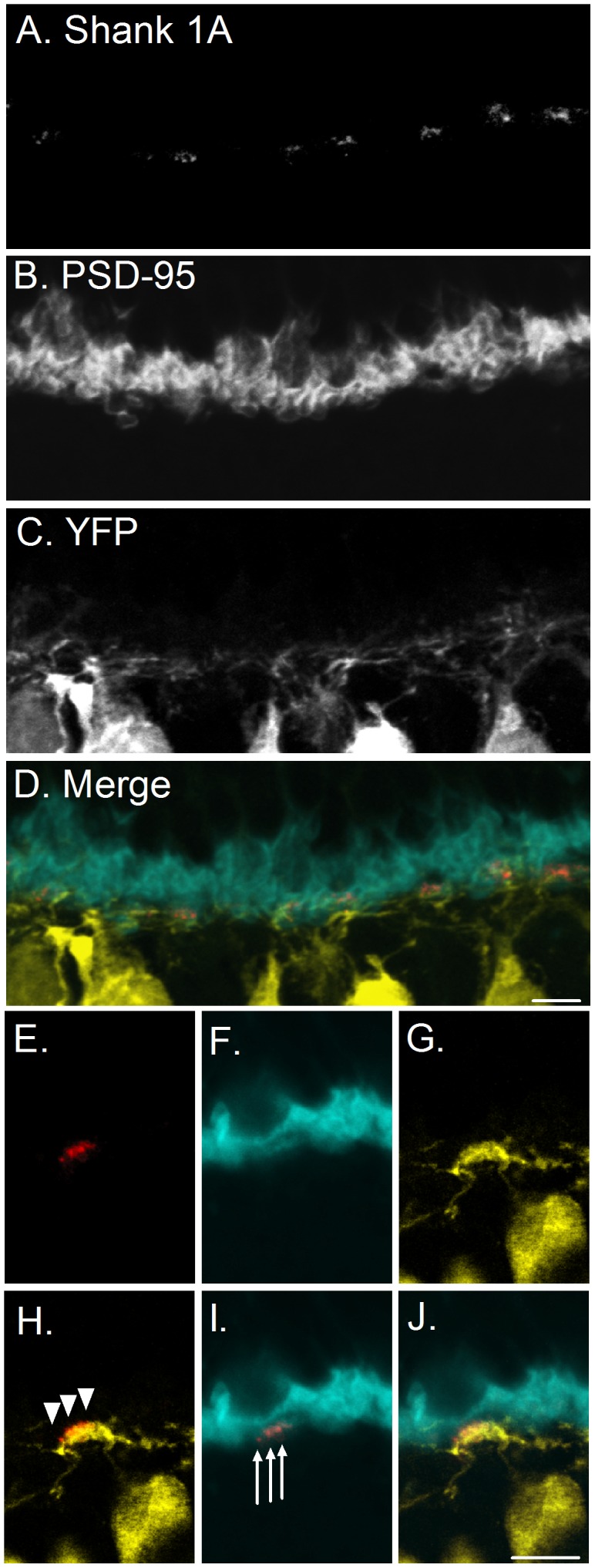
Shank 1A is expressed with PSD-95 labeled photoreceptor terminals in the mouse thy-1.2 YFP 16 line retina. **A–D**: Zoomed confocal region of the OPL immunostained with rabbit anti-Shank1A and mouse anti-PSD-95 antibodies. A. Shank 1A puncta labeling in the OPL in the mouse retina. B. PSD-95 immunolabels rod and cone photoreceptor terminals in the OPL. C. YFP labeled dendrites from cone bipolar cells in the OPL. D. Merged confocal image showing Shank 1A (red), PSD-95 (blue), and YFP (yellow). **E–J**: High magnification single plane confocal images of Shank 1A and PSD-95 in the mouse thy 1.2 YFP 16 line retina (100× objective, N.A. 1.3). E. Shank1 A (red). F. PSD-95 (blue). G. YFP (yellow). H. Merged image of YFP and Shank 1A, YFP and Shank 1A puncta are not co-localized at cone bipolar cell dendrites (see arrowheads, shank fluorescence above YFP dendrite. I. Merged image of Shank 1A and PSD-95, the two immunoreactivities (pink) indicate co-localization of Shank 1a and PSD-95 (see arrows which indicate that Shank 1A is co-localized with PSD-95). Arrows indicate location of Shank 1A immunoractive puncta co-localized with PSD-95. J. Merged image of inset Shank 1A (red), PSD-95 (blue), and YFP (yellow). Primary antibodies were detected using a secondary goat anti-rabbit Alexa 568 IgG for Shank 1A, and a goat anti-mouse Alexa 633 IgG to PSD-95. Scale bar is 5 µm.

PSD-95 showed strong immunolabeling throughout the OPL (Fig. 3). In our hands the PSD-95 antibody typically labeled only the OPL, with very faint or no labeling in the IPL as described previously [31]. To investigate whether Shank 1A is associated with PSD-95 we performed double immunolabeling experiments with antibodies to Shank 1A and PSD-95 on adult YFP-16 mouse vertical retinal sections. We found that Shank 1A puncta were co-localized with PSD-95 in clusters just above the YFP labeled dendrites (see Fig. 3D and J, co-localized signal is the pink colored shank 1A puncta in the OPL). PSD-95 shows a strong signal within the photoreceptor terminal region, and significant overlap of Shank 1A and PSD-95 is generally observed at all areas with Shank 1A expression (Fig. 3D I and J). In contrast, YFP and Shank 1A show no significant overlap of the two signals, with most of the fluorescence out of phase with the YFP dendritic process (Fig. 3H). Moreover, in these experiments we used anti-fluorescent protein antibodies (anti-GFP/anti-YFP) to enhance weakly labeled dendrites to rule out the possibility that the YFP signal is weakly expressed at the dendrites of cone bipolar cells. Shank 1A labeling was absent from any YFP labeled cone bipolar cell dendrites consistent with Shank 1A labeling being confined to photoreceptor terminals in the outer retina. We also confirmed the synaptic localization of all Shank 1A antibodies used in this study with the PSD-95 antibody in mouse cerebellar cultures (data not shown) as described previously [9], [24]. These findings indicate that Shank 1A is clustered with PSD-95 at photoreceptor synapses, and not associated with YFP cone bipolar cell processes.

### Shank 1A is expressed at ribbon synapses in the OPL

**Figure 4:**
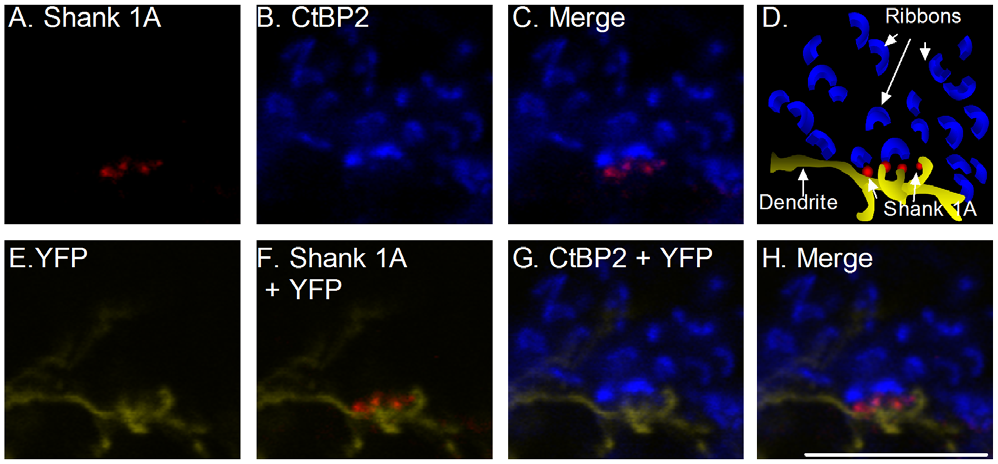
Shank 1A immunoreactivity is not associated with the synaptic ribbon protein. High magnification zoom of a region in the OPL. **A–H**: Confocal images from the mouse thy-1.2 YFP 16 line immunolabeled with Shank 1A and CtBP2 (a homologue of RIBEYE), a marker of synaptic ribbons in mammalian retina. A. Shank 1A (red). B. CtBP2 (blue). C. A combined fluorescence image showing Shank 1A (red) and CtBP2 (blue). D. A schematic illustrating the zoomed confocal image in H, showing that the Shank 1A puncta (red) is located distal and adjacent to the tips of the YFP dendrite (yellow), and are surrounded by CtBP2 labeled synaptic ribbon protein structures (blue). E. YFP (yellow) F. A combined fluorescence image showing Shank 1A (red) and YFP (yellow). G. A combined fluorescence image showing CtBP2 (blue) and YFP (yellow). H. A combined triple labeled image showing Shank 1A (red), CtBP2 (blue), and YFP (yellow). Scale bar = 10 µm.

The relationship between Shank 1A and the synaptic ribbon was determined by evaluating the distribution of the synaptic protein RIBEYE, which is a splice variant of the transcription factor CtBP2 [32], [33], and Shank 1A in the OPL at photoreceptor terminals. In vertical retinal sections, CtBP2 labeling was characterized by multiple horseshoe shaped structures within the OPL (Fig. 4), and diffuse labeling of nuclei in all cell layers, with Shank 1A labeling restricted to the OPL and IPL. The immunoreactivities of the two proteins did not overlap at the cone photoreceptor-cone bipolar cell dendrites; instead, it appears that Shank 1A puncta were expressed just distal to the YFP cone bipolar cell dendrites, with the horseshoe shaped CtBP2 immunolabeled ribbon structures surrounding Shank 1A in the cone pedicle (Fig. 4 C and H). Shank 1A and VGLUT1 immunostaining overlapped in cone pedicles (see Fig. S2), which is consistent with a presynaptic site of expression for Shank 1A. VGLUT1 immunoreactivity generally fills the synaptic terminal, which is indicative of vesicles present throughout the photoreceptor terminal (Figs. S2B, D, F, and G); however, the most intense VGLUT1 immunolabeling always appeared to be adjacent to or co-localized with the synaptic ribbon (data not shown). Shank 1A puncta showed the most intense labeling at the base of the VGLUT1-containing cone terminal (Figs. S2 D and G). However, without further ultrastructural analysis it is difficult to draw any conclusions regarding Shank 1A and VGLUT1 localization, still it is clear that Shank 1A is present within VGLUT1-containing photoreceptor terminals.

### Shank 1A is restricted to cone photoreceptor terminals

**Figure 5:**
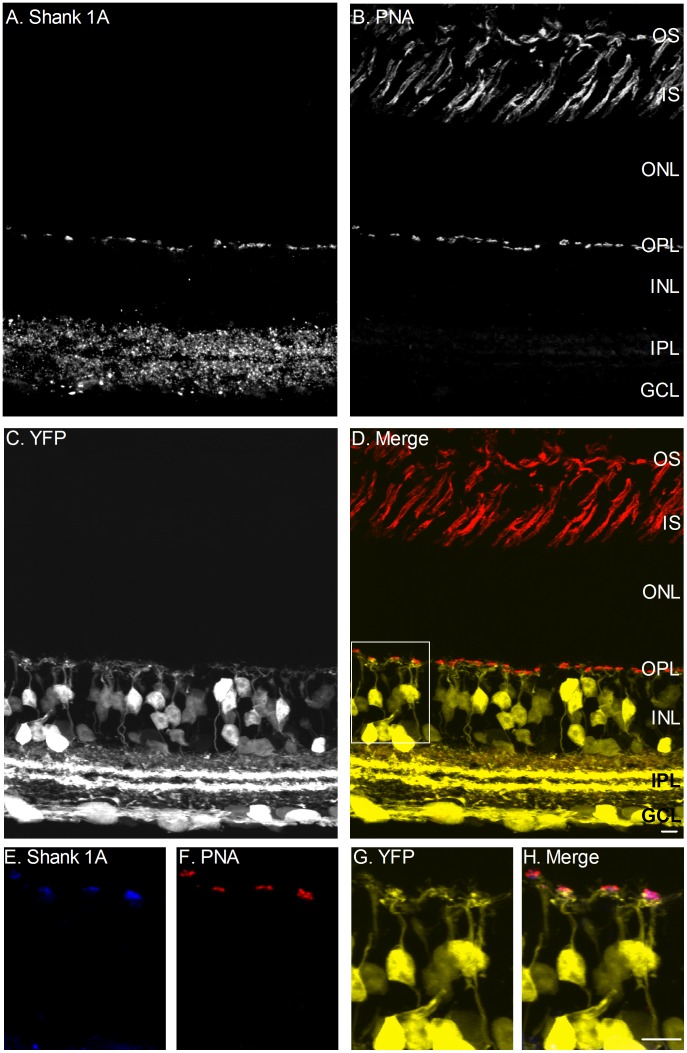
Shank 1A immunoreactivity is co-localized with the lectin PNA (peanut agglutinin) in photoreceptor terminals. **A–D**: Combined labeling of Shank 1A, PNA, and YFP in the mouse thy-1.2 YFP 16 line vertical retinal section. A. Shank 1A labels both the OPL and IPL. The immunoreactive puncta in the OPL are indicative of cone photoreceptor labeling. B. PNA conjugated rhodamine labels the inner and outer segments of cone photoreceptors, and cone photoreceptor terminals in the OPL. C. YFP fluorescence is present in bipolar, amacrine, and ganglion cells. D. Combined triple fluorescence channel image of PNA (red), Shank 1A (blue), and YFP (yellow). A box is drawn of a region in the OPL and high magnification images are shown in E–H. **E–H**: High magnification zoom of a region in the OPL from D. E. Shank 1A (blue). F. PNA (red). G. YFP labeled cone bipolar cells and their dendrites (yellow). H. A combined triple labeled fluorescent image showing that Shank 1A (blue) is expressed at the same site as PNA (red) above the YFP cone bipolar cell dendrites (yellow). OS = outer segment, IS = inner segment, ONL = outer nuclear layer, OPL = outer plexiform layer, INL = inner nuclear layer, IPL = inner plexiform layer, and GCL = ganglion cell layer. Scale bars is 10 µm.

Since expression of Shank 1A is likely restricted to cone terminals we tested whether the cone marker, peanut agglutinin (PNA) conjugated to rhodamine, co-localized with Shank 1A. Vertical sections were immunostained with Shank 1A and then treated with PNA-conjugated rhodamine (Fig. 5). PNA labeled the base of cone terminals (red) and inner and outer segments of cones in the outer retina (Fig. 5 B and D). In the OPL, the PNA signal (red) overlapped strongly with the Shank 1A signal (blue), suggesting that PNA and Shank 1A were expressed at the same site (inset of box in 5D see Fig. 5E–H). This can be observed in the high magnification confocal scans of the OPL (see inset, Fig. 5E–H). Since PNA can bind to the extracellular side of the cone terminal, we wanted to determine whether Shank 1A immunofluorescence is not obscured by the close proximity of the YFP dendrites. To test this possibility we dissociated solitary cone photoreceptors and YFP labeled bipolar cells from YFP-16 mouse retinas and labeled cones with Shank 1A, PSD-95, and PNA, and YFP cone bipolar cells with Shank1A and PNA (Fig. S3). Shank 1A, PSD-95, and PNA were localized to the terminal (Fig. S3 A–D), and both Shank1A and PNA were absent from the dendrites of YFP cone bipolar cells (Fig. S3 E, F, and H), providing additional support that Shank 1A is present at cone photoreceptor terminals.

**Figure 6:**
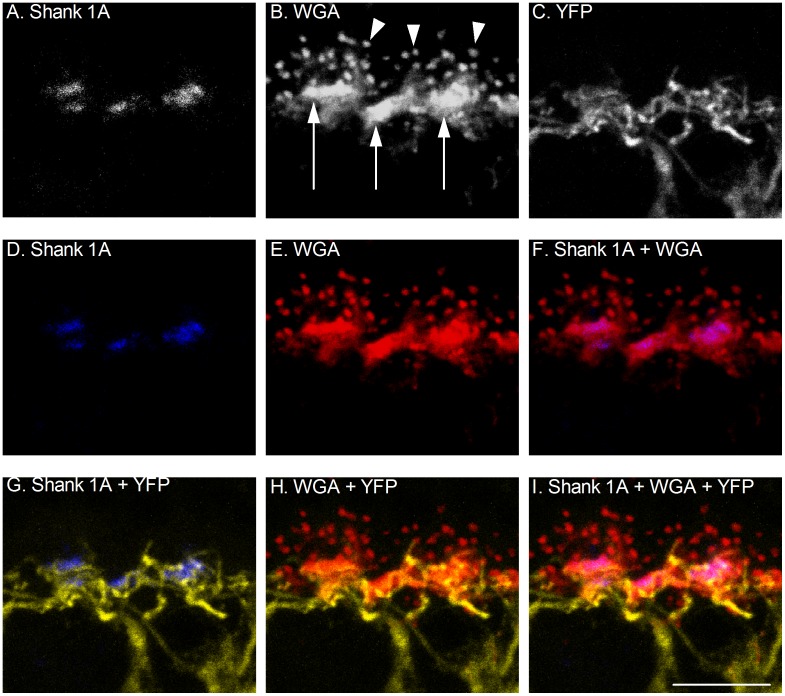
Shank 1A immunoreactivity co-localizes with the lectin WGA at cone photoreceptor terminals. **A–C**: A. Shank 1A B. WGA labeling at the OPL. Arrowheads indicate WGA rod spherule labeling, and arrows indicate the location of WGA labeling of cone pedicles. C. YFP cone bipolar cell dendrites. **D–F**: Shank 1A (blue) and WGA (red) co-localize at cone terminals in the OPL. G–I: YFP cone bipolar cell dendrites (yellow) synapse with WGA (red) labeled cone terminals, which cradle Shank 1A immunoreactive puncta (blue). Scale bar is 10 µm.

To definitively test whether any rod photoreceptor terminals possess Shank 1A, double labeling with Shank 1A and wheat germ agglutinin (WGA) were performed. WGA has been used as a marker of rod and cone terminals in the mouse retina [34], [35], and WGA is localized distal to the dendritic tips of PKCα labeled rod bipolar cell dendrites (Fig. S4), illustrating that cone pedicles are proximal to distal rod spherules in the OPL. Therefore, we wanted to ascertain whether any Shank 1A signal was present at rod terminals. Fig. 6 illustrates a high magnification confocal image of WGA and Shank 1A labeling at the dendrites of a YFP labeled mouse cone bipolar cell. Rod terminals labeled with WGA had a punctate appearance and measured about 0.5 µm diameter, and they were located in the OPL distal to YFP cone bipolar cell dendrites (see arrowheads Fig. 6B). Cone terminals labeled with WGA are characterized as large rectangular blobs that measured about 2–3 µm in length (see arrows Fig. 6B) and they were cradled by YFP cone bipolar cell dendrites (Fig. 6H). WGA labeled both rod and cone photoreceptor terminals (Fig. 6), and co-localized with Shank 1A in cone pedicles (Fig. 6F–I), showing that Shank 1A is absent from rod terminals, and restricted solely to cone terminals.

**Figure 7:**
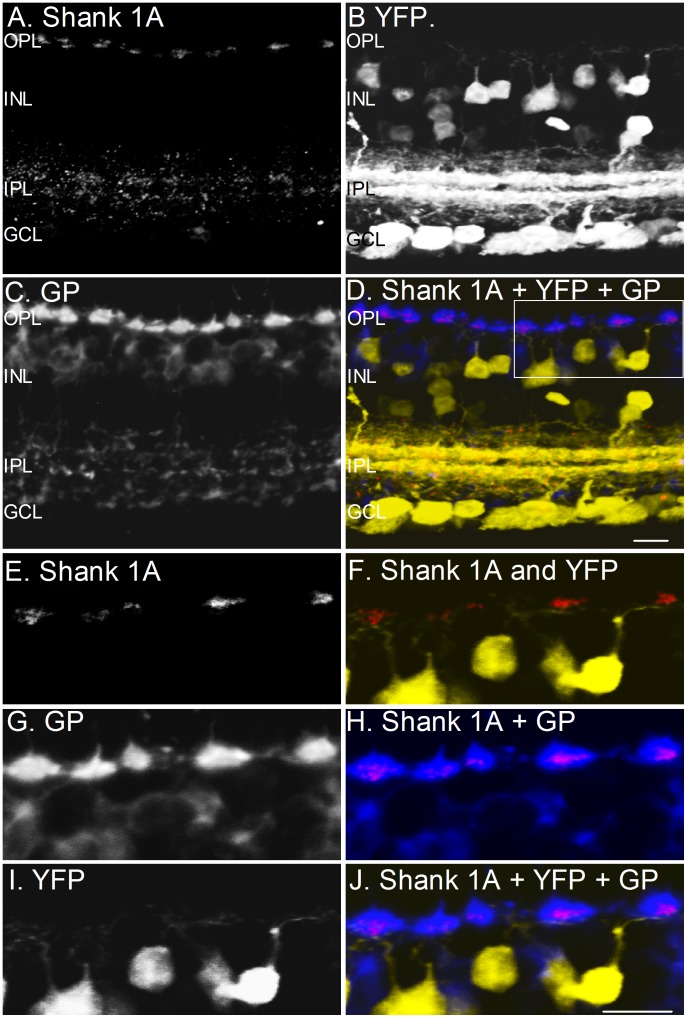
Shank 1 immunoreactivity is expressed solely within cone terminals of the mouse thy-1.2 YFP 16 line retina. **A–D**: Shank 1A is co-localized with glycogen phsophorylase (cone photoreceptor marker). **A–D**: Combined labeling of Shank 1A, glycogen phosphorylase, and YFP in a vertical retinal section. A. Shank 1A labels both the OPL and IPL. The immunoreactive puncta in the OPL are indicative of cone photoreceptor labeling. B. YFP fluorescence is present in bipolar, amacrine, and ganglion cells. C. Glycogen phosphorylase (GP), a cone photoreceptor marker strongly labels the cone terminals with faint labeling of bipolar cell bodies and their axons. D. Combined triple label fluorescence image of Shank 1A (red), YFP (yellow), and glycogen phosphorylase (GP) (blue), and. A box is drawn of a region in the OPL and high magnification images are shown in E–J. **E–J**: High magnification zoom of a region in the OPL from the inset above in Fig. 7D. E. Shank 1A. F. Merge of Shank 1A (red) and YFP (yellow), showing that Shank 1A (red) is expressed above the cone bipolar cell dendrites (yellow). G. Glycogen phosphorylase (GP). H. Merge of Shank 1A (red) and Glycogen phosphorylase (GP), the two immunoreactivities (pink) indicate co-localization of Shank 1a and Glycogenphosphorylase (GP). I. YFP labeled cone bipolar cells and their dendrites. J. Combined image of Shank 1A (red), glycogen phosphorylase (blue), and YFP cone bipolar cell dendrites (yellow). OPL = outer plexiform layer, INL = inner nuclear layer, IPL = inner plexiform layer, and GCL = ganglion cell layer. Scale bar is 10 µm.

To determine whether cone photoreceptor terminals possess Shank 1A, double labeling experiments with anti-glycogen phosphorylase and Shank 1A were performed on mouse YFP retinal sections. Glycogen phosphorylase selectively labels cone photoreceptors in the mouse retina [37], [38]. Fig. 7 shows both low (Fig. 7A–D) and high (Fig. 7E–J) magnification images demonstrating the localization of Shank 1A immunostaining within glycogen phosphorylase-immunoreactive cone terminals. Cone terminals labeled with glycogen phosphorylase show consistent co-localization with Shank 1A immunoreactive puncta (Fig. 7D, H, and J), however YFP dendrites don't co-localize with shank 1A puncta (Fig. 7F) and generally don't overlap or contact Shank 1A puncta at cone terminals arguing that Shank 1A is localized entirely within the cone pedicle (Figs. 7H and J).

## Discussion

This is the first report of Shank 1 expression in the mammalian retina revealing Shank 1 immunoreactivity within both synaptic layers of the retina. More importantly, there is a differential distribution of Shank 1A among photoreceptors in the mouse outer retina. Shank 1A is localized exclusively at cone pedicles and it is absent from rod spherules. Shank 1A immunostaining demonstrated strong overlap with the lectin, PNA, and it was found in the terminal region of glycogen phosphorylase labeled cone photoreceptors providing strong evidence that Shank 1A is restricted to the cone pedicle. In addition, PSD-95 is co-localized together with Shank 1A at cone photoreceptor terminals, suggesting a potential role for Shank 1A. We also found that Shank 1A immunoreactivity is not co-localized with the synaptic ribbon protein RIBEYE/CtBP2, suggesting that Shank 1A is not associated with synaptic ribbons, but it is likely to be associated with PSD-95 near the base of the synaptic terminal. The emerging role of structural signaling proteins, like Shank 1A and PSD at the cone synapse will be considered further below, however, it is conceivable that these protein interactions might provide novel ways to target proteins to the synapse (e.g., Ca^2+^ channels), modulate cone synaptic plasticity, and perhaps influence transmitter release from cones.

### Shank 1A expression in the OPL and IPL

The finding that Shank 1A is clustered in cone terminals within PSD-95 (Fig. 3), but absent from YFP cone bipolar cell dendrites, strongly supports a presynaptic localization of the protein in the cone terminal. More importantly, deletion of the Shank 1 gene completely abolished all Shank 1A immunoreactivity in the outer and inner retina (Fig. 2), demonstrating that the observed immunoreativity is specific to Shank 1A. Also, it is important to note that the synaptic ribbon and PSD scaffolding organization is not disrupted by the deletion of Shank 1 gene, since there is no difference in the PSD-95 and CtBP2 immunolabeling between wild type and shank1 (-/-) mice (Fig. S1). This doesn't rule out the possibility that signaling in the outer retina has not been altered in cones by this genetic deletion of the shank 1 gene. Support for this conclusion comes from a previous study examining nyctalopin gene deletion in mouse retina [36], the expression pattern of both synaptic and signaling proteins (PSD-95, etc.) appeared to be normal in the absence of nyctalopin, it is possible that one or more may not be functional. Alternatively, a lack of function could be due to mislocalization that is not discernible at the light-microscope level and requires ultrastuctural analysis, which may be the case for Shank 1 and is beyond the scope of this study.

Shank 1A expression was absent from both horizontal cells (data not shown) and cone bipolar cells (Figs. 1, 4, 5, and 6) which is in contrast to Shank 2 expression in dendrites of ON bipolar cells and horizontal cell processes [39]. However, it is clear from our findings that unlike Shank 2, Shank 1A immunoreactivity is restricted solely to cone photoreceptor terminals and lies at or near the plasma membrane just above the YFP-expressing cone bipolar cell dendrites (Figs. 5 and 7). In addition, Shank 1A is likely to form a tight association with PSD-95 via GKAP in cone terminals, similar to previous reports of the relationship of Shank 1A and PSD-95 in hippocampal neurons [3], [8], [9], [21], and it is possible that any one of the four GKAP family members (GKAP/SAPAP1-4) could link Shank 1A to PSD-95 in cones. However, we were unable to test this relationship due to the lack of anti-GKAP antibodies that immunostain retinal tissue. Shank 1A unlike other members of the Shank family (2 and 3), are restricted solely to neurons in the mature central nervous system [8], [25], [26] and characterized by interactions with Ca_V_1.3 (α_1D_) L-type Ca^2+^ channels [4], [22], [23]. This raises the possibility that Shank 1A could influence L-type Ca^2+^ channels expressed at mammalian cone photoreceptor synapses [40], [41], [42], [43], [44], [45].

In the inner retina, Shank 1A-immunoreactive puncta were homogeneously distributed throughout the IPL, and were present in amacrine and ganglion cell processes (Fig. 1G–L). Similar to Shank 2, Shank 1A in the IPL is likely to form a postsynaptic complex composed of an NMDA receptor (NR1), PSD-95 and GKAP [8]. This is supported by the findings that Shank 2 is associated with the NR2A subunit of the NMDA receptor [39], and that PSD-95 and the NR1 subunit of the NMDA receptor, co-localize at many postsynaptic sites in the IPL [1]. In addition, Shank 1A was absent from bipolar cell dendrites but it is likely to be present at post-synaptic sites in the inner retina, suggesting a role for Shank 1A at inner retinal PSDs. The only other Shank family member that has been studied in the retina to date is Shank 2/ProSAP1 [39], [46]. The presence and localization of the Shank 2 protein with postsynaptic glutamate receptors suggest that Shank 2 has a functional role at postsynaptic sites, where it assembles with glutamate receptors and links them to the cytoskeleton and downstream signaling pathways.

### Shank 1A expression at cone but not rod ribbon synapses

Double labeling of Shank 1A with PNA, WGA, and glycogen phosphorylase demonstrated that Shank 1A expression was restricted to cone terminals (Figs. 5, 6, 7). Interestingly, the VGLUT1 signal overlapped with Shank 1A; however, VGLUT1 identifies synaptic vesicles and is diffusely distributed throughout the entire cone terminal (Fig. S2). Therefore, it is likely that we observe putative co-localization due to the large number of synaptic vesicles present within the cone terminal, and we observe overlap of these two signals at the light microscopy level. A more detailed analysis at the ultrastructural level is needed to determine the relationship of VGLUT1 and Shank 1A expression in cone terminals. It is possible that Shank 1A could interact with the underlying cytoskeleton to target vesicles to the synaptic ribbon or plasma membrane, but this is purely speculative without a detailed ultrastructural analysis. More interesting, synaptic ribbon protein CTBP2/RIBEYE does not co-localize with Shank 1A at the cone terminal (Fig. 4), but instead Shank 1A is located near the base of the ribbon (Fig. 4 C–H), likely adjacent to the plasma membrane where it could form a complex with PSD-95 (Fig. 3). Shank 1A is likely to be associated with the local actin-based cytoskeleton through the N-terminal ankyrin repeats or short proline-rich regions, based on previous reports in central neurons [8], [24]. Support for a functional role of Shank 1A in the regulation of synaptic architecture in cones comes from studies at central synapses, where Shank 1 has been shown to regulate dendritic spine morphology, and dysregulation of Shank 1 synthesis results in abnormal spine development [47], and loss of the functional architecture at synapses in Alzheimer's disease [48]. It is also possible that Shank 1A mediates the targeting of cytoskeletal proteins, receptors and ion channels (e.g., Ca^2+^ or IP_3_R channels) to the plasma membrane in cones. Support for this idea comes from studies in hippocampal neurons, where expression of Shank 1A, alone or together with other structural signaling proteins (e.g., Homer), corresponded with the appearance of IP_3_Rs and the accumulation of complete endoplasmic reticulum (ER) cisternae in the spines of hippocampal neurons [7], providing evidence for Shank 1A in regulating Ca^2+^ homeostasis in neurons. Since Ca^2+^ signaling is a hallmark of neurotransmission in photoreceptors, Shank 1A may have similar role in Ca^2+^ regulation at the cone pedicles.

### Functional significance of Shank 1A at cone photoreceptor terminals

Since the discovery of PSD proteins at photoreceptor terminals [1], the functional role of these proteins has been an enigma. The PSD is thought to be the prime target for postsynaptic plasticity, and the entry of Ca^2+^ ions into the postsynaptic compartment [21], [49], [50], [51]. Ca^2+^ entry through Ca^2+^-permeable glutamate receptors is considered to be the key signal for the induction and regulation of plastic changes at postsynaptic sites [52], [53], [54]. However, Shank proteins target multiple types of proteins, including Ca^2+^ channels [4], [22], [23]. In mammalian retina, photoreceptors express high voltage-activated dihydropyridine (DHP)-sensitive, L-type Ca^2+^ channels [Ca_V_1.3 (α_1D_) or Ca_V_1.4 (α_1F_)], which regulate transmitter release (L-glutamate) from photoreceptors in the retina [40], [42], [44], [45], [55], [56], [57]. Evidence that cone and rod photoreceptors express different subtypes of L-type Ca^2+^ channels comes from numerous functional and anatomical studies in both non-mammalian and mammalian species. For example, in mammals some cones express predominately the Ca_V_1.3 (α_1D_) pore forming subunit [42], [57], whereas rods express predominately the Ca_V_1.4 (α_1F_) pore forming subunit [42], [55], [56]. Since, Shank 1A has been shown to interact with both PSD-95 and L-type Cav1.3 Ca^2+^ channels in other preparations [4], [22], [23], it is possible that Shank 1A could interact with Cav1.3 Ca^2+^ channels at the cone synapse to influence transmitter release, although very intriguing this is highly speculative and will need to be investigated further in the future. Taken together, the discovery of Shank 1A expression at cone photoreceptor terminals sheds new light on the understanding of PSDs and their associated proteins that might influence differences in photoreceptor signaling in the mammalian retina.

## Materials and Methods

### Ethics Statement

All animal procedures were approved by UCLA Animal Research Committee, the UCLA Division of Laboratory Animal Medicine, and conducted according to the *Guide for the Care and Use of Laboratory Animals*, published by the National Institutes of Health (NIH Publication No. 85–23, Revised 1996).

### Tissue Preparation

The retinas used for this study were obtained from 12 week old or order mice of either sex, and included the following mouse strains: C57BL/6; *Thy*-1.2 YFP mice 16 Jrs [*thy*-1.2 YFP-16 line (B6.Cg-Tg(*thy1*-YFP)16Jrs), The Jackson Laboratory, Bar Harbor, ME] or Shank 1 (-/-) knockout mice (129SvJae/C57BL/6). The Shank 1 (-/-) knockout mice were generated as previously described [29]. Briefly, chimeric mice were produced by injecting targeted ES cell clones into C57BL/6 blastocysts, and heterozygous offspring were backcrossed into C57BL/6 and 129SvJae strains. The Shank1 (-/-) knockout animals used for experiments in this study were in a 129SvJae/C57BL/6 hybrid genetic background.

All studies followed the guidelines prescribed by the UCLA Animal Research Committee, the UCLA Division of Laboratory Animal Medicine, and the U.S. National Institutes of Health/National Eye Institute. Adult mice were housed in standard cages at ∼23°C on a 12-hr/12-hr light/dark cycle. Mice were deeply anesthetized with either a lethal dose of Nembutal (80–90 mg/kg) or isoflurane (30% v/v) in a covered glass container (volume: 1L). The animal was decapitated and the eyes were removed. The eyes were opened along the ora serrata, the cornea, lens, and vitreous body was removed and the eyecups were immersion-fixed in 4% paraformaldehyde (PFA) in 0.1 M phosphate buffer (PB; pH 7.4) for 15 to 30 minutes at room temperature. The eyes were then cryoprotected in 25% sucrose overnight at 4°C. Prior to cutting the tissue with a cryostat the retina was washed with 0.1 M PB and embedded in Tissue-Tek OCT compound (Sakura Finetek Inc., Torrance, CA) and rapidly frozen with dry ice or liquid nitrogen. Cryostat sections of the retina were cut at 12–15 µm, mounted on to gelatin-coated slides, air dried, and stored at −20°C.

### Retinal Dissociations

Adult YFP-16 mice 10 to 12 weeks of age were deeply anesthetized with a lethal dose of Nembutal (80–90 mg/kg). The animal was decapitated and the eyecup is removed. The eyes are opened along the ora serrata, the cornea, lens, and vitreous body is removed. Each retina was isolated from the eyecup in Ca^2+^-free Mg^2+^-free Hanks Balanced Salt Solution (HBSS) (Invitrogen, Carlsbad, CA). The retina was transferred to a flask containing papain (10–15 U/ml) and DL-cysteine (1 mg/10 mls) in Ca^2+^-free Mg^2+^-free HBSS. For photoreceptor isolation the retinas were agitated on an orbital shaker at 37°C in a humidified CO2 incubator for 15 minutes. For bipolar cells the retinas were agitated on an orbital shaker at 37°C in a humidified CO2 incubator for 35 minutes. The retinas were carefully washed three times in Dulbecco's Modified Eagle Medium (DMEM, Invitrogen, Carlsbad, CA) containing 10% fetal bovine serum (FBS, Invitrogen, Carlsbad, CA) and DNase (50 U/ml, Worthington). For photoreceptor isolation the retina was carefully triturated through a 200 µl eppendorf pipette by gently applying suction to the photoreceptor side of the retinal pieces and then and plated onto concanavalin A (1 mg/ml) (Sigma, St. Louis, MO) coated coverslips and allowed to settle for 15–30 min. For bipolar cell isolation the retina was carefully triturated through a fire-polished fine bore Pasteur pipette and plated onto concanavalin A (1 mg/ml) coated coverslips. Bipolar cells were allowed to settle for at least 30 minutes. Dissections were usually performed under room light or dim red illumination.

### Antibodies

Three Shank 1 antibodies were used for this study: 1) a rabbit polyclonal antibody raised against the COOH-terminal region of Shank 1A (1∶1000) using the product of pGex4T-1 synamon-16, which is the COOH-terminal region of Shank 1A (a generous gift from Dr. Yukata Hata, Tokyo Medical and Dental University, Tokyo, Japan [24]; 2) a rabbit polyclonal antibody raised against the following sequence SGPIYPGLFDIRSS in the COOH-terminal region of Shank 1A (1∶500–1∶1000) (Catalog No. RA19016, Neuromics, Edina, MN); and 3) a guinea pig polyclonal Shank 1 antibody (1∶20–1∶25) was generated through the use of a glutathione S-transferase (GST)-fusion of the PDZ domain of SSTRIP/Shank 1. For this purpose the corresponding cDNA fragment was cloned into pGEX4T-2, and purification and isolation were performed as described previously [28]. All Shank 1 antibodies produced the same pattern of punctate immunolabeling in the OPL and the inner plexiform layer (IPL) of the mouse retina, and immunostaining was absent in retinas where the Shank 1 gene was deleted (Fig. 2). These antibodies have been previously well characterized in neurons [7], [24], [28]. PSD-95 was identified in photoreceptor terminals with a purified mouse monoclonal antibody raised against the amino acid sequence 353–504 of PSD-95 (1∶500–1∶1000) (Catalog No. 610495, BD Biosciences, San Jose, CA). A rabbit polyclonal antibody was raised against residues 659–672 from the COOH-terminal variable (V5) region of rat protein kinase C α was used to identify rod bipolar cells (1∶200,000) (Catalog No. P4334; Sigma Chemical St. Louis, MO). A guinea pig polyclonal antibody raised against the vesicular glutamate transporter 1 (VGLUT1) was used to identify glutamate-containing photoreceptor and bipolar cell terminals (1∶40,000–1∶80,000) [Catalog No. AB5905; Millipore, Temecula, CA; [58]]. A purified mouse monoclonal antibody raised against the amino acid sequence 361–445 of the C-terminal binding protein 2 (CtBP2) (1∶2000) (Catalog No. 612044; BD Biosciences, San Jose, CA) was used to label synaptic ribbons [32], [33]. A rabbit polyclonal antibody raised against a brain specific sequence of glycogen phosphorylase [59] was used to label cone photoreceptors in the mouse retina (1∶1000) [a generous gift from Dr. B. Hamprecht, University of Tübingen, Tübingen, Germany [37], [38]]. To enhance, the YFP labeling in the dendrites of cone bipolar cells either anti-YFP (1∶500) (Catalog No. ABIN411626; antibodies-online GmbH, Atlanta, GA) or anti-GFP (1∶250) (Catalog No. 06–896; Millipore, Temecula, CA) antibodies were used in some experiments. See Table 1 for a complete list of antibodies used in this study and their sources.

**Table 1:** Antibodies and Lectins.

Antibody/Lectin	Dilution	Host	Immunizing antigen	Remarks	Reference	Source
Shank 1A	1∶1000	Rabbit (IgG)	COOH-terminal region of Shank 1A	Labels cone terminals; Results identical in Western blots and immunohistochemistry to polyclonal Shank 1 (Kreienkamp) and Shank 1A (Neuromics)	Yao et al., 1999	Dr. Yukata Hata, Tokyo Medical and Dental University, Tokyo, Japan
Shank 1A C-terminus	1∶800–1,000	Rabbit (IgG)	raised against the following sequence SGPIYGLFDIRS in the COOH-terminal region of Shank 1A	Labels cone terminals (see above)	-	Neuromics, Edina, Mn (Catalog # RA19016)
Shank 1	1∶20–1∶25	Guinea Pig (IgG)	Generated through the use of a glutathione S-transferase (GST)-fusion of the PDZ domain of SSTRIP/Shank1	Labels cone terminals (see above)	Quitsch et al., 2005	Dr. Hans-Juergen Kreienkamp, Universitätsklinikum Hamburg-Eppendorf, Hamburg, Germany
Protein Kinase C α	1∶200,000	Rabbit (IgG)	raised against residues 659–672 from the COOH-terminal variable (V5) region of rat protein kinase C α	Labels rod bipolar cells	Zhang and Yeh, 1991	Sigma Chemical Corp., St Louis, Mo. (Catalog # P4334)
Vesicular Glutamate Transporter (VGLUT1)	1∶80,000	Guinea pig (IgG)	Peptide sequence GATHSTVQPPRPPPPVRDY from rat VGLUT1	Labels glutamatergic vesicles in photoreceptors and bipolar cell terminals	Johnson et al., 2003; Sherry et al., 2003	Millipore, Temecula, Ca, Catalog # AB5905
C-terminal Binding Protein 2 (CtBP2)	1∶2,000	Mouse (IgG)	recombinant protein consisting of amino acid sequence 361–445 at C-terminal binding protein 2	Labels the synaptic ribbon	Tom Dieck et al., 2005	BD Transduction Laboratories, San Jose, CA Catalog # 612044
Glycogen Phosphorylase	1∶1,000	Rabbit (IgG)	The carboxy-terminal region of glycogen phosphorylase, containing the following peptide sequence were used GVEPSDLQIPPPNLPKD.	Labels cone photoreceptors	Haverkamp et al., 2005; Wässle et al., 2006.	Dr. Hamprecht, University of Tübingen, Tübingen, Germany
PSD-95	1∶1000	Mouse (IgG)	Recombinant rat PSD95	Labels photoreceptor terminals. Characterized previously in Koulen et al., J. Neurosci. 18:10136, 1998.	Blackmon et al., 2000	BD Biosciences, San Jose, CA Catalog No. 610495
Yellow Flourescent Protein (YFP)	1∶500	Rabbit (IgG)	Recombinant YFP expressed from *E. coli*	Selectively labels XFP variants	_	Antibodies-online GmbH, Atlanta, GA, catalog # ABIN411626
Green Flourescent Protein (GFP)	1∶250	Chicken (IgY)	His-tagged green fluorescent protein of *Aequorea victoria*	Selectively labels XFP variants	_	Millipore, Temecula, Ca, Catalog # 06-896
Peanut agglutinin (PNA)	1∶250–1∶500	_	_	Labels cone outer and inner segments and cone terminals	Blanks and Johnson, 1984	Vector Laboratories, Burlingame, CA, Catalog # RL-1072
Wheat Germ Agglutinin (WGA)	1∶1,500	_	_	Labels rod and cone terminals in the outer retina	Fariss et al., 1990; Iwasaki et al., 1992.	Vector Laboratories, Burlingame, CA, Catalog # RL-1022

To check for antibody specificity, controls were prepared by omitting one or two of the three primary antibodies for a triple label immunostaining procedure or one of the two primary antibodies for a double label immunostaining procedure. In these control experiments only the immunoreactivity for the remaining primary antibody and nonspecific background staining were detected, as in the case of single labeling experiments, in which the primary antibody was omitted. All antibodies were tested on mouse retinal tissue as single labeling experiments at least three times to confirm specificity and optimize concentration prior to performing any double or triple labeling experiments to assure specific labeling.

Characterization and evidence for appropriate use of antibodies as cell and synaptic markers are as follows:

1. Anti-Shank 1/Shank 1A: These antibodies label the presynaptic cone terminals in the mouse retina (Fig. 1). Western blot analysis showed that Shank 1 (also known as synamon and SSTRIP) antibodies labeled a series of protein bands with a molecular mass in the range of 240–288 kDa only in brain tissues, and this band was not present in other tissues and regions [24], [30]. The intensity of these bands was weaker in the heterozygote and absent in the homozygous Shank 1-/- knockout mouse brain [29]. Shank 1A immunostaining was absent in retinal sections obtained from Shank 1 -/- knockout mice (Fig. 2) that were immunostained with the three antibodies used in this study.
2. Anti-PSD-95: This antibody is a marker for photoreceptor terminals in the retina [1], [31]. In our hands, labeling was restricted to the OPL, with very faint or no labeling in the IPL, as described previously [31]. This antibody detects a 95-kDa band on Western blots of rodent brain lysate that is blocked by preincubation with the antigen [[60], [61]; manufacturer's data sheet].
3. Anti-PKCα: PKCα is a well-established marker for rod bipolar cells in the retina [62]. This antibody detects an 80-kDa band on Western blots of rat brain that is blocked by preincubation with the antigen, but not by preincubation with corresponding peptides of other PKC isoforms (manufacturer's data sheet, SIGMA-Aldrich).
4. Anti-VGLUT1: VGLUT1 is a marker of glutamatergic terminals of photoreceptor and bipolar cells in the retina [58], [63]. This antibody detects a single band on Western blots of the hippocampus at approximately 67-kDa (manufacturer's data sheet).
5. Anti-CtBP2: This antibody shares sequence homology with RIBEYE a marker of synaptic ribbons in the retina [33]. This antibody detects a ∼50-kDa band of the B-domain of RIBEYE on Western blots [33].
6. Anti-glycogen phosphorylase: This antibody is a cone photoreceptor marker in mouse retina [37], [38]. This is a brain specific antibody that detects a 97-kDa band in brain homogenates on Western blots [59].
7. Anti-YFP: This antibody selectively immunolabels native and denatured forms of GFP and its variants EGFP, YFP, EYFP, and CFP. See manufacture's data sheet.
8. Anti-GFP: This antibody immunostains cells transfected with an expression vector encoding GFP, and the antibody also cross reacts with YFP. See manufacturer's data sheet.

### Lectins

Rhodamine conjugate of wheat germ agglutinin (WGA) (Catalog No. RL-1022; Vector Laboratories, Burlingame, CA) and rhodamine conjugate of peanut agglutinin (PNA) (Catalog No. RL-1072, Vector Laboratories, Burlingame, CA) were used to label rod photoreceptor spherules and cone photoreceptor pedicles, respectively (see Figs. 5, for PNA labeling and Figs. 6 and S4 for WGA labeling; [34], [35], [64]). Lectin conjugated fluorophores were incubated along with secondary antibodies to reveal specific labeling. WGA also weakly labels cell surface membranes of retinal neurons in addition to rod spherules and cone pedicles in the OPL (data not shown).

### Immunohistochemistry

All tissue was labeled using the indirect immunofluorescence technique [65], [66]. Briefly, retinal sections were warmed for 10 minutes at 37°C, and preincubated in a 0.1 M PB mixture containing 10% normal goat serum (NGS) (Invitrogen, Carlsbad, CA), 1% bovine serum albumin (BSA) (Sigma-Aldrich, St. Louis, Mo) and 0.5% Triton-X 100 (Sigma-Aldrich, St. Louis, Mo) for 1 hour. The sections were then incubated in primary antibodies, which were all diluted in 0.1 M PB (pH 7.4) containing 3% NGS, 1% BSA and 0.5% Triton-X 100 overnight at 4°C. The primary antibody/antigen complex was detected using secondary antibodies conjugated to either Alexa 568, Alexa 633, Alexa 647 or Alexa 700 (Invitrogen, Carlsbad, CA). The retinal sections were washed three times for 10 minutes following the antibody incubation with 0.1 M PB to remove any unbound primary or secondary antibody. For double or triple labeling experiments retinal sections were incubated in a mixture of primary antibodies followed by a mixture of secondary antibodies. All slides were allowed to air dry in the dark at room temperature and coverslipped with Prolong Gold anti-fade (Invitrogen, Carlsbad, CA).

Zenon labeling technology was used in some experiments when two primary antibodies with the same serotype were combined in an experiment (e.g., rabbit anti-glycogen phosphorylase and rabbit anti-Shank 1A). Briefly, slides were prepared as described above, during the blocking step; the primary antibodies were prepared using the Zenon labeling kit (Catalog No. Z-25360; Invitrogen, Carlsbad, CA), antibodies were incubated for 5 minutes in a mixture of primary rabbit antibody (IgG) and Alexa conjugated rabbit Fab fragment (molar ratio: 1 primary antibody (rabbit IgG): 3 Alexa Fab fragment). The Fab fragment binds to the Fc portion of the rabbit IgG. In each reaction tube the Alexa conjugated Fab fragment is neutralized by the addition of excess rabbit IgG for 5 minutes (molar ratio: 1 primary antibody: 3 rabbit IgG fragment). In these experiments, Alexa conjugated 568 or Alexa conjugated 633 Fab fragments were used. Following the tissue blocking step both solutions were combined and brought to a total volume of 200–250 µl with a solution containing in 0.1 M PB (pH 7.4) containing 3% NGS, 1% BSA and 0.5% Triton-X 100. The retinal sections were then incubated in this mixture for 30 minutes to 1 hour at room temperature. The sections were then washed 2 to 3 times with 0.1 M PB and 0.1% Tween-20 (Sigma-Aldrich, St. Louis. Mo). To insure proper cross linking of Fab fragments and rabbit primary IgGs a second fixation was performed with 4% PFA for 5 minutes. The retinal sections were then washed 2–3 times with 0.1 M PB with 0.1% Tween-20 and coverslipped. In addition, Zenon labeling technology was used for some antibodies on mouse retinal tissue, where cross-reactivity limitations of the secondary antibody interfered with detection of the primary antibody. Briefly, monoclonal primary antibodies were complexed with Alexa 568-labeled Fab fragments directed against their Fc regions (Catalog No. Z-25006; Invitrogen, Carlsbad, CA).

### Confocal Microscopy

Images of retinal sections were acquired using a Zeiss Laser Scanning Microscope 510 META (Zeiss, Thornwood, NY) with Plan Apochromat 100×/1.40 Oil DIC objective, Plan Apochromat 63×1.4 NA oil objective, Plan Neofluar 40×1.3 NA oil objective, or a C-Apochromat 40×1.2 NA water objective. To identify fluorescent signals, different lasers were used for excitation, for YFP the 488 nm argon laser line was used, for Alexa 568, the 543 nm HeNe laser line was used; and for Alexa 633, Alexa 647, or Alexa 700 the 633 nm HeNe laser line was used. During acquisition of signals from double-labeled or triple labeled specimens, the scans were collected sequentially to prevent spectral bleed-through. Specific band-pass filters were used to achieve proper separation of signals (for double labeling 488/505–530, 543/560LP; for triple labeling, 488/505–530, 543/560–610, 633/650LP). To reduce any further bleed-through of spectral signals, linear unmixing was employed in some scans. Most images were acquired at a resolution of 2048×2048, and in some cases 1024×1024, as either 12-bit or 8-bit signals. To increase the signal-to-noise, images were averaged online (e.g. n = 4) and the scan speed and photo multiplier detector gain were decreased. Most confocal images were acquired at an approximate optical thickness of 0.5 µm or 1.0 Airy unit. For projections typically 8–10 optical sections were acquired with an average total thickness of 5 µm and compressed for viewing. Some images have been deconvolved to remove out of focus fluorescence using an iterative deconvolution algorithm using Zeiss LSM 510 Meta software ver. 4.2 (Zeiss Ltd, Thornwood, NY). Digital confocal images were saved as Zeiss. LSM files and final publication quality images were exported in the. TIFF format as 300 dpi. All images were processed and adjusted for brightness and contrast using Adobe Photoshop 7.0 or CS3 Extended (Adobe Systems Inc., Mountain View, CA).

## Supporting Information

Figure S1 PSD-95 and CtBP2 immunolabeling in the Shank 1 (-/-) mouse retina. **A–C**: A. CtBP2 labeled the OPL and IPL with faint labeling of cell bodies in the INL and GCL (red) B. PSD-95 labeled the OPL (red) C. Merged image of CtBP2 and PSD-95 immunolabeling. **D–E**: High magnification zoom of the OPL. D. CtBP2 (red) E. PSD-95 (green) F. CtBP2 (red) and PSD-95 (green) merged image. PSD-95 structures cluster around CtBP2 horseshoe shaped structures in the OPL. OPL = outer plexiform layer, INL = inner nuclear layer, IPL = inner plexiform layer, and GCL = ganglion cell layer. Scale bar is 10 µm.
(TIF)

Figure S2 Shank 1A immunoreactivity is present within photoreceptor VGLUT terminals. **A–C**: High magnification confocal scan of a photoreceptor terminal in the OPL immunolabeled with Shank 1A and VGLUT1 antibodies. A. Shank 1A. B. VGLUT1. C. YFP dendrite. D. Shank 1A (red) is expressed within the VGLUT1 (blue) labeled photoreceptor terminal. E. Shank 1A puncta (red) is located distal to the YFP dendrite (yellow) in the photoreceptor terminal. F. VGLUT1 (blue)-containing photoreceptor terminal located distal to the YFP dendrite (yellow) in the OPL. G. A combined triple labeled fluorescent image showing that Shank 1A (red) is expressed within the VGLUT1 (blue)-containing cone terminal and above the YFP cone bipolar dendrite (yellow). A schematic diagram (last panel in the first row) of the panel G illustrates the expression of Shank 1A at the cone photoreceptor-cone bipolar cell terminal. With VGLUT1 (blue) and the YFP dendrite (yellow). OPL = outer plexiform layer. Scale bar is 10 µm.
(TIF)

Figure S3 Mouse Isolated cone photoreceptor and YFP cone bipolar cell. All images are shown with their respective bright field DIC image overlaid to illustrate the structures of the cells. **A–D**: Isolated cone photoreceptor. A. Shank 1A labeling (green) in the terminal of an isolated cone bipolar cell. B. PSD-95 labeling (blue) in the terminal of an isolated cone photoreceptor. C. PNA (red) labels the outer and inner segments (IS), the soma, and the terminal of the cone photoreceptor. D. Merged image of Shank1A, PSD-95, and PNA. In panel D the outer segment (OS), inner segment (IS), terminal and soma are identified by arrows. **E–H**: Isolated YFP cone bipolar cell. E. Shank 1A (no labeling present) F. PNA (no labeling present) G. YFP (yellow) H. Merged image showing only the YFP fluorescence. Scale bar is 5 µm.
(TIF)

Figure S4 Wheat germ agglutinin (WGA) labels the terminals of rod and cone photoreceptors in the OPL. A. WGA conjugated rhodamine. B. PKCα, a marker for rod bipolar cells, labels the dendrites, the soma, and axon. C. Combined WGA (red) and PKC (blue) image illustrating that WGA puncta sit above the dendrites of the PKCα labeled rod bipolar cell. See higher magnification image of the boxed region on the right in C. Arrows indicate WGA puncta and the location of rod photoreceptor terminals. D. Combined WGA (red) and YFP (yellow) image illustrating that WGA also labels cone terminals in the OPL. Cone labeled WGA puncta are larger than the rod puncta and contact YFP cone bipolar cell dendrites which are located below the rod terminal region. See higher magnification image of the boxed region on the right in D. Arrowheads indicate WGA puncta and the location of cone photoreceptor terminals. E. Combined triple label fluorescent image of WGA (red), PKCα (blue) and YFP (yellow). Scale bar is 10 µm.
(TIF)
