# Supplementary figures and images for: Association of Shank 1A Scaffolding Protein with Cone Photoreceptor Terminals in the Mammalian Retina
Source: PLoS One. 2012 Sep 12;7(9):e43463. doi: 10.1371/journal.pone.0043463 (PMC3440378; doi:10.1371/journal.pone.0043463)

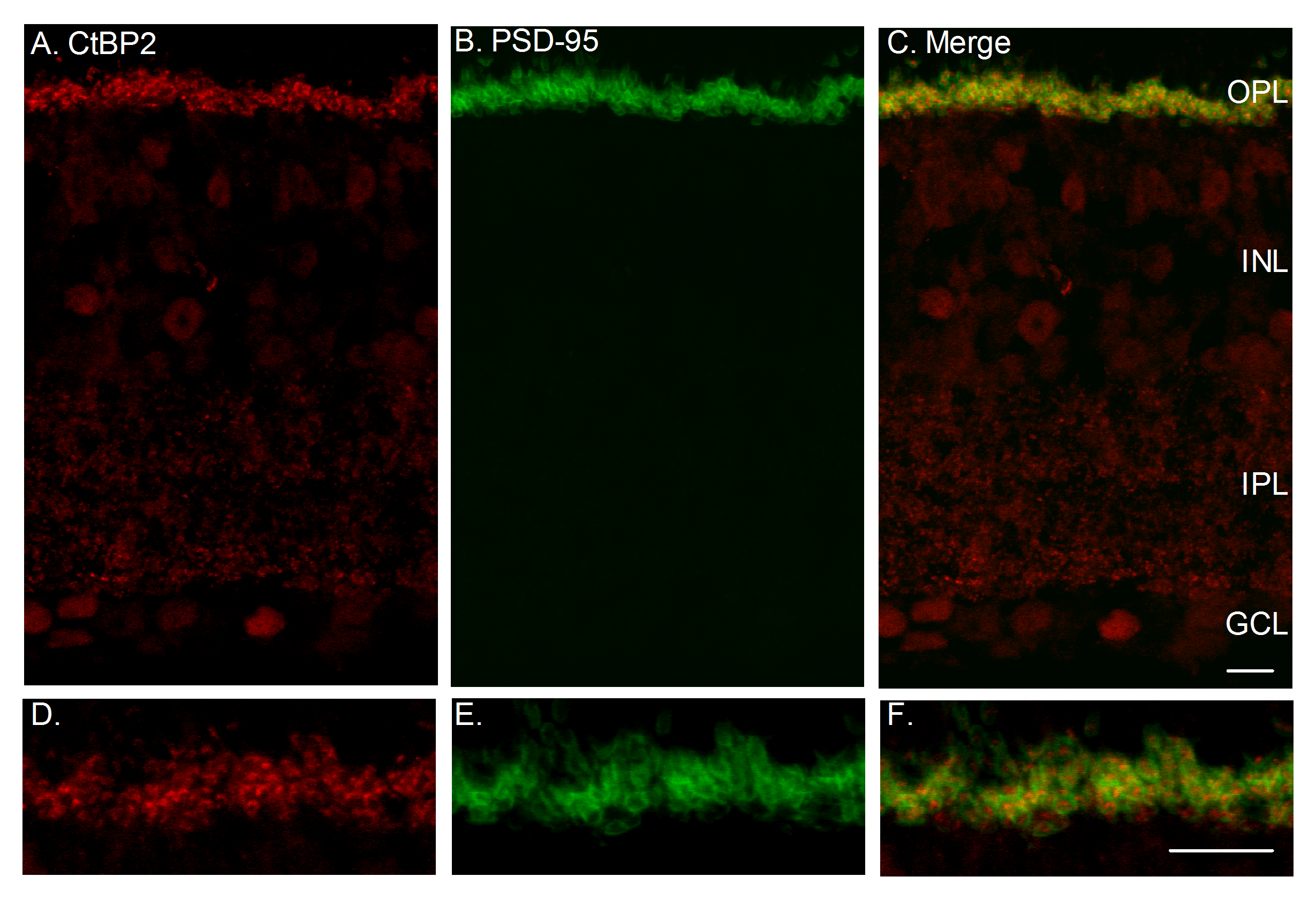

Supplement: Figure S1 — PSD-95 and CtBP2 immunolabeling in the Shank 1 (-/-) mouse retina. A–C: A. CtBP2 labeled the OPL and IPL with faint labeling of cell bodies in the INL and GCL (red) B. PSD-95 labeled the OPL (red) C. Merged image of CtBP2 and PSD-95 immunolabeling. D–E: High magnification zoom of the OPL. D. CtBP2 (red) E. PSD-95 (green) F. CtBP2 (red) and PSD-95 (green) merged image. PSD-95 structures cluster around CtBP2 horseshoe shaped structures in the OPL. OPL = outer plexiform layer, INL = inner nuclear layer, IPL = inner plexiform layer, and GCL = ganglion cell layer. Scale bar is 10 µm. (TIF) [file pone.0043463.s001.tif]

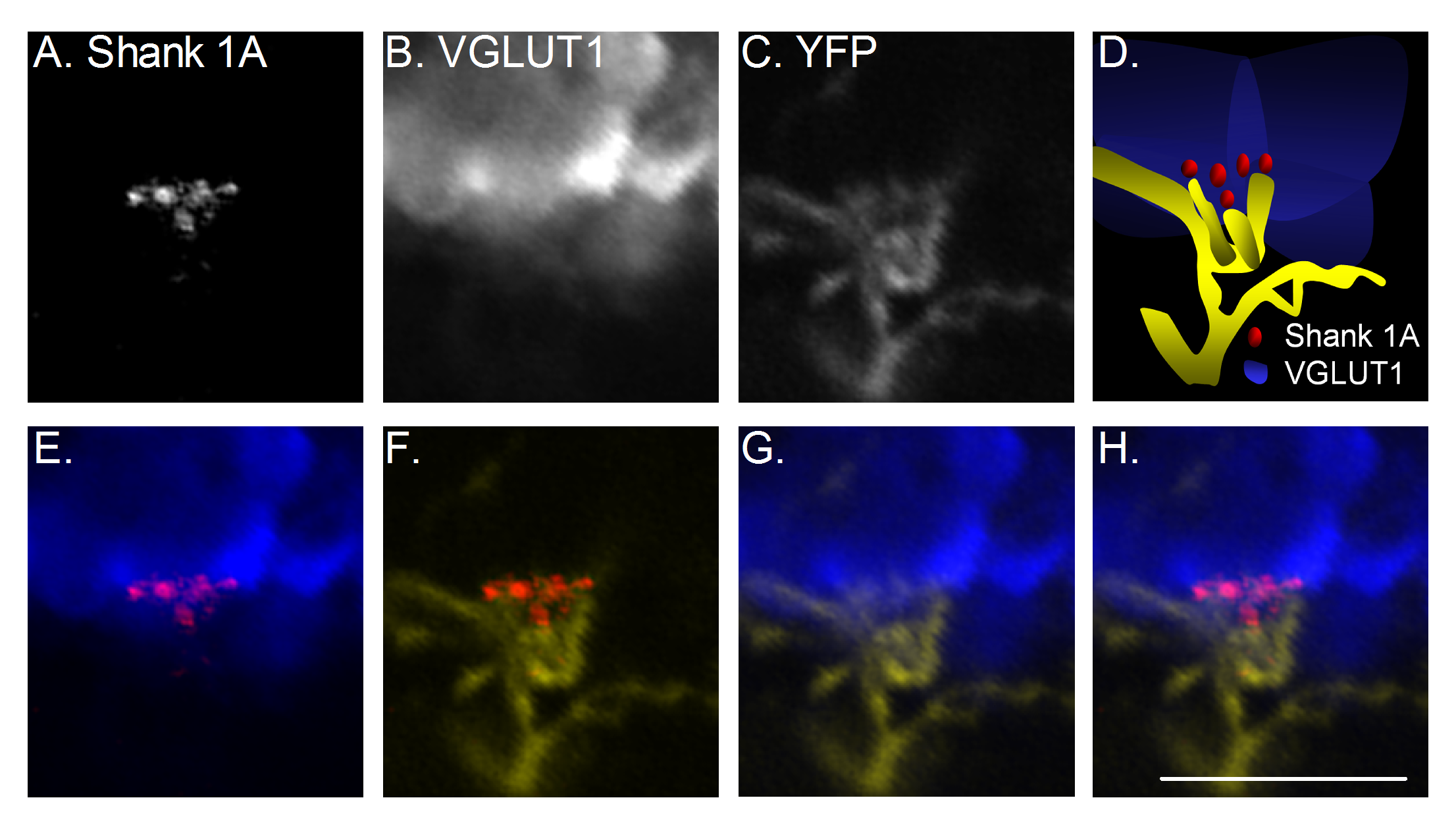

Supplement: Figure S2 — Shank 1A immunoreactivity is present within photoreceptor VGLUT terminals. A–C: High magnification confocal scan of a photoreceptor terminal in the OPL immunolabeled with Shank 1A and VGLUT1 antibodies. A. Shank 1A. B. VGLUT1. C. YFP dendrite. D. Shank 1A (red) is expressed within the VGLUT1 (blue) labeled photoreceptor terminal. E. Shank 1A puncta (red) is located distal to the YFP dendrite (yellow) in the photoreceptor terminal. F. VGLUT1 (blue)-containing photoreceptor terminal located distal to the YFP dendrite (yellow) in the OPL. G. A combined triple labeled fluorescent image showing that Shank 1A (red) is expressed within the VGLUT1 (blue)-containing cone terminal and above the YFP cone bipolar dendrite (yellow). A schematic diagram (last panel in the first row) of the panel G illustrates the expression of Shank 1A at the cone photoreceptor-cone bipolar cell terminal. With VGLUT1 (blue) and the YFP dendrite (yellow). OPL = outer plexiform layer. Scale bar is 10 µm. (TIF) [file pone.0043463.s002.tif]

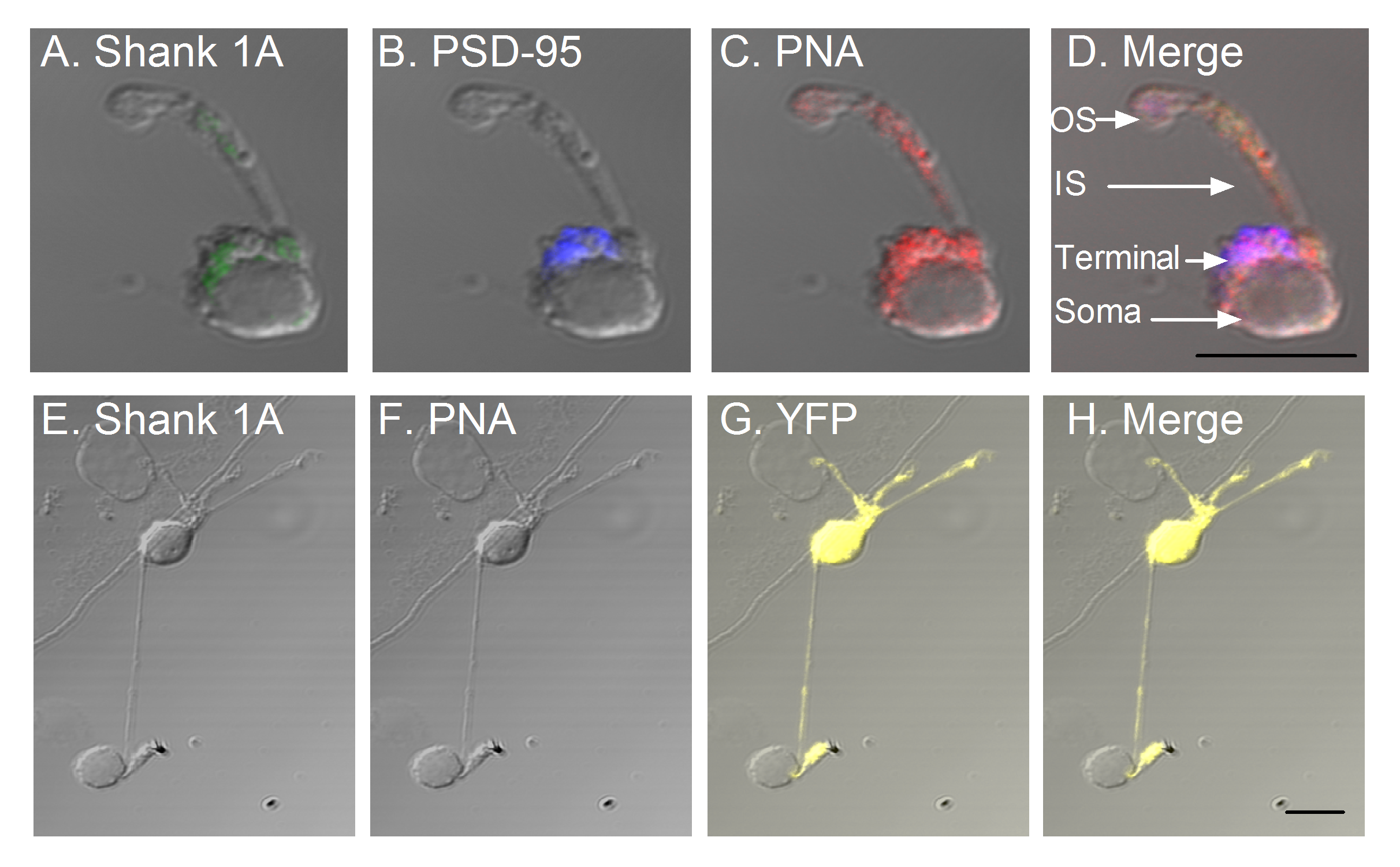

Supplement: Figure S3 — Mouse Isolated cone photoreceptor and YFP cone bipolar cell. All images are shown with their respective bright field DIC image overlaid to illustrate the structures of the cells. A–D: Isolated cone photoreceptor. A. Shank 1A labeling (green) in the terminal of an isolated cone bipolar cell. B. PSD-95 labeling (blue) in the terminal of an isolated cone photoreceptor. C. PNA (red) labels the outer and inner segments (IS), the soma, and the terminal of the cone photoreceptor. D. Merged image of Shank1A, PSD-95, and PNA. In panel D the outer segment (OS), inner segment (IS), terminal and soma are identified by arrows. E–H: Isolated YFP cone bipolar cell. E. Shank 1A (no labeling present) F. PNA (no labeling present) G. YFP (yellow) H. Merged image showing only the YFP fluorescence. Scale bar is 5 µm. (TIF) [file pone.0043463.s003.tif]

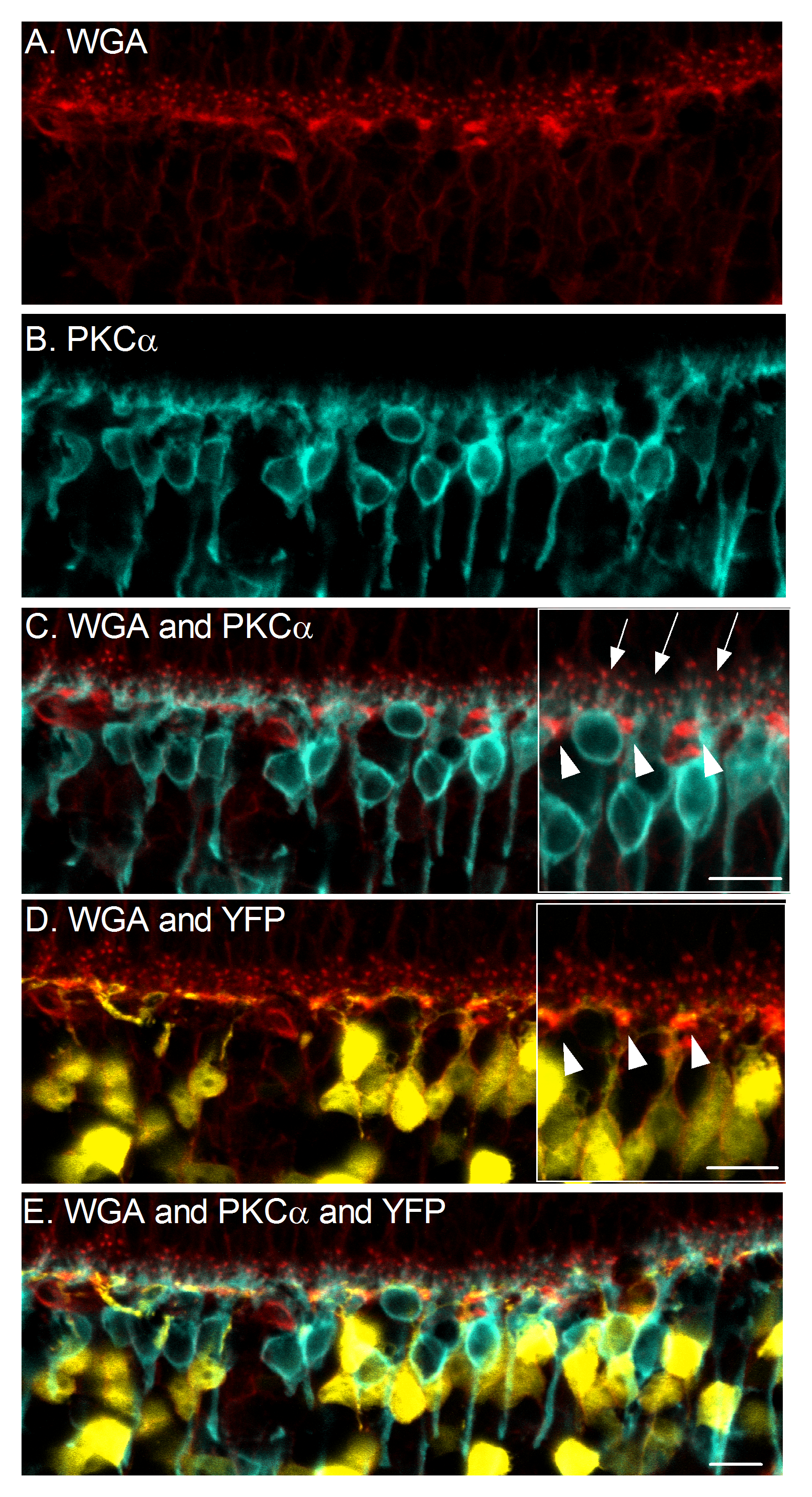

Supplement: Figure S4 — Wheat germ agglutinin (WGA) labels the terminals of rod and cone photoreceptors in the OPL. A. WGA conjugated rhodamine. B. PKCα, a marker for rod bipolar cells, labels the dendrites, the soma, and axon. C. Combined WGA (red) and PKC (blue) image illustrating that WGA puncta sit above the dendrites of the PKCα labeled rod bipolar cell. See higher magnification image of the boxed region on the right in C. Arrows indicate WGA puncta and the location of rod photoreceptor terminals. D. Combined WGA (red) and YFP (yellow) image illustrating that WGA also labels cone terminals in the OPL. Cone labeled WGA puncta are larger than the rod puncta and contact YFP cone bipolar cell dendrites which are located below the rod terminal region. See higher magnification image of the boxed region on the right in D. Arrowheads indicate WGA puncta and the location of cone photoreceptor terminals. E. Combined triple label fluorescent image of WGA (red), PKCα (blue) and YFP (yellow). Scale bar is 10 µm. (TIF) [file pone.0043463.s004.tif]
